# Treatment of Cattle Manure by Anaerobic Co-Digestion with Food Waste and Pig Manure: Methane Yield and Synergistic Effect

**DOI:** 10.3390/ijerph17134737

**Published:** 2020-07-01

**Authors:** Gahyun Baek, Danbee Kim, Jinsu Kim, Hanwoong Kim, Changsoo Lee

**Affiliations:** School of Urban and Environmental Engineering, Ulsan National Institute of Science and Technology (UNIST), 50 UNIST-gil, Eonyang-eup, Ulju-gun, Ulsan 44919, Korea; ghbaek@psu.edu (G.B.); danbee1008@unist.ac.kr (D.K.); penguin0529@unist.ac.kr (J.K.); khw3670@unist.ac.kr (H.K.)

**Keywords:** anaerobic co-digestion, biochemical methane potential, cattle manure, response surface analysis, synergy index

## Abstract

The management of cattle manure (CM) has become increasingly challenging because its production continues to rise, while the regulations on manure management have become increasingly stringent. In Korea, most farms produce CM as a dry mixture with lignocellulosic bedding materials (mainly sawdust), making it impractical to treat CM by anaerobic digestion. To address this problem, this study examined whether anaerobic co-digestion with food waste (FW) and pig manure (PM) could be an effective approach for the treatment of CM. The batch anaerobic digestion tests at different CM: FW: PM mixing ratios showed that more methane was produced as the FW fraction increased, and as the CM fraction decreased. The response surface models describing how the substrate mixing ratio affects the methane yield and synergistic effect (methane yield basis) were successfully generated. The models proved that the methane yield and synergistic effect respond differently to changes in the substrate mixing ratio. The maximum 30-day methane yield was predicted at 100% FW, whereas the maximum 30-day synergy index was estimated for the mixture of 47% CM, 6% FW, and 47% PM (total solids basis). The synergy index model showed that CM, FW, and PM could be co-digested without a substantial loss of their methane potential at any mixing ratio (30-day synergy index, 0.89–1.22), and that a possible antagonistic effect could be avoided by keeping the FW proportion less than 50%. The results suggest that co-digestion with PM and FW could be flexibly applied for the treatment and valorization of CM in existing anaerobic digestion plants treating FW and PM.

## 1. Introduction

The management of livestock manure has become an increasing concern for the livestock industry [1]. Approximately 40 Mt of cattle manure (CM), 5 Mt of pig manure (PM), and 2 Mt of chicken manure are produced every day worldwide [2]. Livestock manure has been traditionally managed by direct application to agricultural land and open composting, causing environmental problems such as soil/groundwater contamination and eutrophication. Anaerobic digestion (AD), which converts organic matter into biogas, is a proven technology to treat high-strength organic wastes, including livestock manure, and has been widely applied [3]. In Europe, more than 18,000 AD plants are in operation, and they produced a total of 63.5 TWh energy out of biogas in 2018 [4]. In the United States, more than 2100 AD plants, including 254 livestock manure digesters, most of which treat CM, are in operation [5].

Energy recovery through AD is an attractive option for the treatment and valorization of organic- and nutrient-rich CM. However, CM has some characteristics that limit its AD, for example, the C/N ratio (5–8) that is significantly lower than the suitable level for stable AD (15–30), the high concentration of toxic free ammonia, and the presence of other potential inhibitors to methanogenesis [6,7]. To overcome the limitations of CM as an AD substrate, many previous studies have attempted co-digestion with other waste biomass, such as food waste (FW) [8,9], agricultural residues [10,11], and other livestock manures [12,13]. Co-digestion is a simple and effective strategy to improve the properties of the mixed substrate for efficient AD by adjusting the carbon/nutrient balance, increasing the buffering capacity, and diluting the concentration of inhibitors [14]. However, co-digestion can sometimes result in an antagonistic effect, depending on the characteristics of the co-substrates [15], and, therefore, the proper selection of co-substrates and their mixing ratio is critical for successful co-digestion [16]. A previous study on the co-digestion of FW and PM reported an antagonistic effect attributable to the inhibition of methanogenesis by excessive nutrients [17]. Another study reported that an antagonistic effect occurred in the mixture of livestock manure and fruit/crop residues because of the low pH (4.16) and rapid fermentation of the latter [18].

CM produced in Korea has a high content of poorly biodegradable lignocellulosic matter because of the widespread use of plant-based bedding materials (mainly sawdust) in cattle sheds. This characteristic further lowers the digestibility of CM and hampers the implementation of AD for CM management in Korea. The annual CM production is more than 22 Mt in Korea and continues to increase [19]. Therefore, an effective method for managing CM is urgently needed, as the regulations on manure management are becoming more stringent. The present study examined the possibility of treating CM through co-digestion with FW and PM, which are among the largest organic waste streams in Korea. The positive effects of co-digesting CM or PM with FW at an appropriate mixing ratio have been reported in many studies, whereas research on the co-digestion of CM and PM or all three is relatively scarce. Ternary mixtures of CM, FW, and PM with different mixing ratios were evaluated for methane potential. The effects of different substrate characteristics and compositions on the methane yield (Y_M_) and synergy index (SI) were investigated using response surface analysis (RSA). The findings of this study provide a reference for the implementation and operation of anaerobic co-digestion processes for the management of CM in Korea.

## 2. Materials and Methods

### 2.1. Preparation of Inoculum and Substrates

Digestate collected from a mesophilic biogas plant co-digesting FW and PM was used as the inoculum in the biochemical methane potential (BMP) experiments. The total solids (TS) and volatile solids (VS) contents of the inoculum sludge were 19.1 g/L and 9.2 g/L, respectively, while the volatile suspended solids content was 7.2 g/L. CM for the BMP tests was prepared by mixing equal weights of five manure samples collected from different cattle pens (thee beef and two dairies; cattle were fed with hay and concentrates) from different farms, to yield a representative CM sample. PM in slurry form was taken from a commercial pig farm. FW, consisting mainly of cooked rice and smaller amounts of flour products, soup, vegetables, and meat, was collected from a cafeteria in the Ulsan National Institute of Science and Technology (Ulsan, Korea) and ground into a slurry using a household blender. The characteristics of each substrate are shown in Table 1.

### 2.2. Analytical Methods

Solids were measured following the protocols in the Standard Methods for the Examination of Water and Wastewater [20]. Chemical oxygen demand (COD), total nitrogen (TN), and total phosphorus (TP) were measured spectrophotometrically using the HS-COD-MR, HS-TN(CA)-H, and HS-TP-H kits (HUMAS, Daejeon Korea), respectively, according to the manufacturer’s instructions. Volatile fatty acids (VFAs, C_2_–C_7_) were measured using a 7820A gas chromatograph (Agilent, Santa Clara, CA, USA) equipped with a flame ionization detector and an Innowax column (Agilent, Santa Clara, CA, USA). The samples for analyzing soluble COD and VFAs were prepared by filtration through a syringe filter with 0.45-μm pore size. Biogas composition was analyzed using a 490 Micro GC system (Agilent, Santa Clara, CA, USA) equipped with dual thermal conductivity detectors coupled to a CP-Molsieve 5Å and a CP-PoraPLOT U column (Agilent, Santa Clara, CA, USA), respectively. The pH and alkalinity measurements were performed with an Orion 3-Star pH meter (Thermo Scientific, Waltham, MA, USA) and an Orion Total Alkalinity Test kit (Thermo Scientific, Waltham, MA, USA) following the manufacturer’s instructions. The C, H, O, N, and S contents (VS basis) were determined using a Flash 2000 elemental analyzer (Thermo Scientific, Delft, The Netherlands). The carbohydrate content was measured as the glucose equivalent by the phenol-sulfuric acid method. The lipid content was analyzed using a Soxtec solvent extraction system (ST 255, Foss, UK). The total Kjeldahl nitrogen (TKN) and ammonia concentrations were determined according to the Kjeldahl method [20]. The protein concentration was calculated by multiplying the concentration of organic nitrogen (i.e., TKN–ammonia nitrogen) by 6.25. The crude fiber was quantified using a FiberCap 2021/2023 system (Foss, Hillerød, Denmark). All analyses were performed at least in triplicate.

### 2.3. Biochemical Methane Potential Test

Thirteen substrate mixtures at different mixing ratios (TS basis) were prepared according to a ternary mixture design (Table 2). All substrate mixtures were diluted to 10 g VS/L, and a 250-mL BMP bottle was filled with 100 mL each of the inoculum and a substrate mixture (i.e., 1 g-VS substrate in a 200-mL working volume). Fourteen BMP runs, including the inoculum-only control to correct for the endogenous methane production, were performed in triplicate (i.e., 42 trials in total). The prepared BMP bottles were purged with nitrogen gas for 1 min to remove oxygen and sealed with a gas-tight rubber stopper. The bottles were then incubated at 35 °C with intermittent manual shaking (once a day) for 30 days while monitoring the biogas production and composition. The volume of produced biogas was measured by inserting the needle of a gas-time syringe through the rubber stopper into the headspace of the BMP bottles. The volume of biogas displaced into the syringe was read after the headspace pressure reached equilibrium with the ambient atmospheric pressure and then corrected to standard conditions (0 °C and 1 atm). The composition of biogas collected from each bottle during the 30-day incubation period was determined by gas chromatography (see Section 2.2 for detailed analytical information).

Y_M_ (methane production per unit mass of substrate) and SI (the observed-to-expected Y_M_ ratio) were determined for each substrate mixture and compared with mono-digestion based on the BMP results. The expected Y_M_ of a mixture was estimated from the proportion of each substrate in the mixture and the mono-digestion yields of individual substrates. Therefore, an SI above 1 indicates synergism, and below 1 indicates antagonism.

### 2.4. Response Surface Analysis

Response surface analysis (RSA) is a mathematical tool for describing the combined effects of multiple independent variables on a dependent variable and for approximating the response of the dependent variable within the experimental design boundary (Table 2). The fractions of CM, FW, and PM in the substrate mixtures (from 0 to 1) were used as independent variables to estimate the responses of Y_M_ and SI. RSA was performed by a sequential procedure of collecting experimental data, constructing polynomial equations, and evaluating the model adequacy. Increasingly complex polynomials were fitted to the experimental data to model the response surfaces of Y_M_ and SI. Backward stepwise regression was applied to select the most suitable response surface model. The Design Expert 7 software (Stat-Ease, Minneapolis, MN, USA) was used to generate the experimental matrix and conduct the RSA calculations for model selection.

## 3. Results and Discussion

### 3.1. Characteristics of the Substrates

CM showed a significantly lower VS/TS ratio (75.9%) compared with FW (95.4%) and PM (80.0%), and the total COD and VS concentrations were the highest in CM (Table 1). These results reflect the large presence of bedding materials mixed in CM, as also indicated by the higher crude fiber content of CM than that of FW and PM (>1.8-fold, VS basis). Lignocellulosic biomass is a complex mixture of organic (cellulose, hemicellulose, lignin, etc.) and inorganic (ash) substances, and an increase in the number of fibrous compounds can lead to an increase in ash content [21]. Additionally, the application of hydrated lime to cattle bedding for disinfection purposes (personal communication with the farm owners) appears to have contributed to the high fixed solids content of CM. Note that the VS/TS ratio of the CM used in this study is significantly lower than the values reported in the literature for other countries (79.9–87.8%) [9,10,22,23,24,25,26]. This difference is likely due to the fact that CM mixed with bedding material stays on the floor of pens generally for six months or longer before being treated by composting in Korea, unlike in many European countries and the United States where wet raw manure is usually scraped out. The unique characteristics of the CM rich in the lignocellulosic matter are also reflected in its significantly higher TS content (31.0%) and C/N ratio (24.3) than those reported for CM in other countries (TS, 8.0–22.2%; C/N ratio, 9.0–17.2) [22,26,27,28,29,30,31]. Too low moisture content leads to reduced fluidity and biodegradability, thereby causing difficulties in the digester operation, and co-digestion with high-moisture substrates is a simple solution to this problem [32].

PM showed a remarkably higher VFA concentration than FW and CM (over five-fold), with acetate, propionate, and butyrate (9.3, 5.2, and 7.8 g COD/kg, respectively) as the major VFA components (Table 1). A high VFA concentration can disturb the balance between acid-forming and acid-consuming reactions and thus inhibit methanogenesis [33,34]. PM also contained a significantly higher level of toxic free ammonia nitrogen than the other substrates (>3-fold), consistent with the literature [35,36]. FW had the most favorable characteristics for AD in terms of organic content and nutrient balance, in accordance with the fact that the suitability of FW as a substrate for AD has been widely demonstrated [37]. The high carbohydrate content of FW (59.3% of VS) can be beneficial for hydrolysis because carbohydrates are generally more readily degradable than proteins and lipids [38]. However, too rapid fermentation can lead to digester souring and even failure [39].

### 3.2. Biochemical Methane Potential Test Results

**Figure 1:**
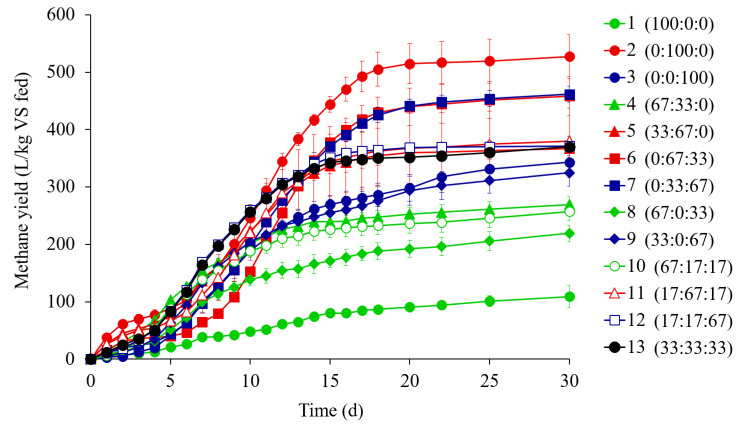
Cumulative methane production in the batch co-digestion tests with cattle manure (CM), food waste (FW), and pig manure (PM). Curves are labeled with the corresponding run numbers (percentage mixing ratio of CM: FW: PM on a total solids basis). Error bars indicate standard deviations (*n* = 3). Symbol colors represent the composition of substrate mixtures (total solids basis): green for high-CM runs (≥67% CM), red for high-FW runs (≥67% FW), blue for high-PM runs (≥67% PM), and black for the center point run with an equiproportional mixture of CM, FW, and PM.

The methane production profiles of the BMP runs varied with the substrate mixing ratio (Figure 1). The FW mono-digestion run (Run 2) produced the most methane (527.5 L/kg VS), followed by the runs of the FW/PM mixtures (Runs 6 and 7), whereas the CM mono-digestion run (Run 1) produced the least (109.2 L/kg VS). Y_M_ generally tended to increase as the FW fraction increased, and as the CM fraction decreased in the substrate mixtures. All high-FW mixtures (≥67% FW) yielded >350 L CH_4_/kg VS, and all high-CM mixtures (≥67% CM) yielded <270 L CH_4_/kg VS. The high-FW runs showed faster initial methane production with no lag, which could be attributed to the rapid utilization of readily bioavailable organics in FW, as reflected in its high soluble-to-total COD ratio (Table 2). These results agree with those of previous reports that methane production was greater when substrate mixtures contained more FW and less CM in their co-digestion [8,40]. Note that the mixtures with higher VS/TS ratios (i.e., higher organic matter fraction) showed higher Y_M_ values (Spearman *p* < 0.01). This suggests that the biodegradable organic content of the substrate mixture was critical, although not exclusive, factor influencing co-digestion efficiency.

**Figure 2:**
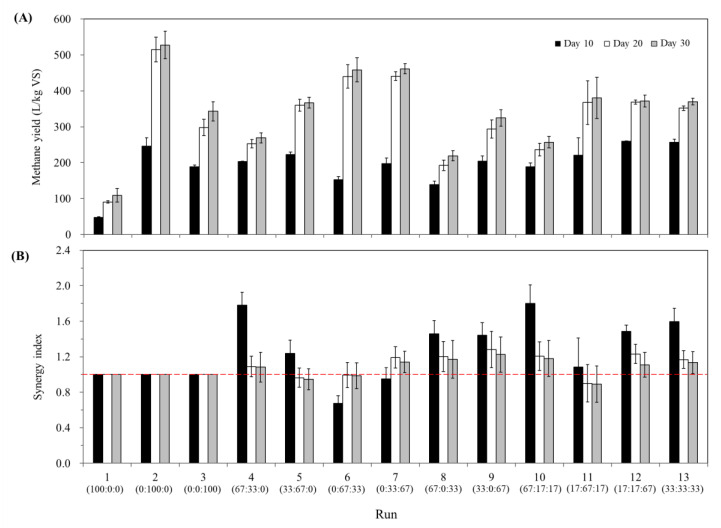
Methane yields (**A**) and synergy indices (**B**) determined on days 10, 20, and 30 of the BMP experiments. The CM: FW: PM mixing ratio (total solids basis) for each BMP run is given in parentheses below the corresponding run number. The red dashed line in panel (**B**) indicates where the synergy index is 1. Error bars indicate standard deviations (*n* = 3).

Figure 2 compares the Y_M_ and SI values for the early (10 days), middle (20 days), and total (30 days) incubation periods among the BMP runs. Y_M_ increased with incubation time regardless of the substrate mixing ratio, and over 90% of the 30-day Y_M_ was produced within 20 days in most runs (except Runs 1, 3, and 8). This result indicates that the 30-day incubation was sufficient for the BMP runs to reach a plateau in methane production. Interestingly, in contrast to Y_M_, SI decreased during the incubation in all runs except the FW/PM co-digestion runs (Runs 6 and 7). This result suggests that the synergistic effect of the co-digestion was more pronounced in the early period of incubation (i.e., 10-day SI), which seems to reflect the accelerated initiation of AD due to the increased levels of readily utilizable organics to stimulate microbial activity by mixing with co-substrates [16]. For the FW/PM co-digestion runs, SI was below 1 on day 10 and increased to around 1 or higher on days 20 and 30. This observation suggests that co-digesting FW and PM led to a transient antagonistic effect during the early incubation, which could be related to the high free ammonia and VFA concentrations in PM and the high availability of rapidly fermentable organics in FW (Table 1).

The 10-day SI varied significantly among the BMP runs from 0.67 to 1.80, whereas the 20- and 30-day SI values ranged from 0.90 to 1.28 and from 0.89 to 1.22 (around 1), respectively. This result indicates that the beneficial effect of co-digestion (i.e., improved substrate properties) was primarily on the reaction rate, particularly during the early period of incubation, rather than the ultimate methane yield in the BMP runs. Such an acceleration was particularly pronounced in the co-digestion runs of high-CM mixtures, suggesting that mixing with more readily utilizable co-substrates promoted microbial growth and enzyme production to facilitate the hydrolysis of slowly biodegradable organics in CM [41].

### 3.3. Response Surface Modeling Results

To more comprehensively evaluate the effects of the substrate mixing ratio on Y_M_ and SI, the response surface models were generated based on the 30-day BMP results. Each experimental data set was fitted with linear to cubic polynomials to model the response surface.

Based on the statistical significance and simplicity, a reduced cubic model was chosen as the most suitable model to estimate the 30-day Y_M_ response surface (Equation (1)):

*Y_Y_*_30_ = 116.8*X_C_* + 516.1*X_F_* + 343.8*X_P_* + 185.2*X_C_X_P_* + 96.6*X_F_X_P_* − 460.8*X_F_X_P_*(*X_F_* − *X_P_*) (1)

where *Y_Y_*_30_ is the predicted 30-day Y_M_, and *X_C_*, *X_F_*, and *X_P_* are the fractions of CM, FW, and PM in the substrate mixtures (between 0 and 1), respectively. The constructed model showed an excellent approximation of the response surface (*R*^2^ > 0.97, *p* < 0.05), and the coefficient of variation was fairly low (Table 3). The adequacy of the precision value, which measures the range of the model response relative to the average prediction error, was much greater than 4, which is the minimum for an adequate response surface model [42]. The model was also checked for the normality assumption by generating a normal probability plot of residuals for the regression equation. The residuals were scattered about a straight line without any pattern (data are not shown), indicating that the model errors follow a normal distribution. Consequently, the obtained model proved adequate for approximating the 30-day Y_M_ response surface.

**Figure 3:**
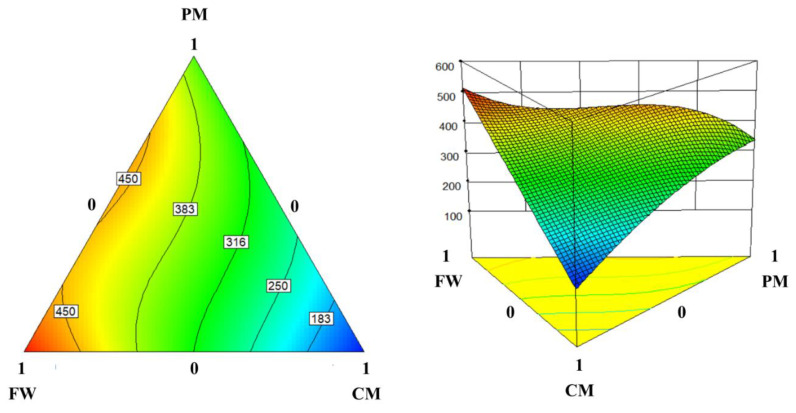
Two- and three-dimensional response surface plots illustrating the effect of the substrate mixing ratio on the 30-day methane yield. Contour colors represent the levels of model response: blue for low and red for high responses.

The Y_M_ response surface plot shows that the predicted response increases with an increasing FW fraction and decreases with an increasing CM fraction in the substrate mixtures (Figure 3). The maximum and minimum model outputs were found at the FW and CM vertices, respectively. Correspondingly, the linear mixture of the *X_C_*, *X_F_*, and *X_P_* has the most significant effect on the model predictions (*p* < 0.0001; Table 4). This result indicates that the 30-day Y_M_ was determined primarily by the linear effects of the fractions of CM, FW, and PM in the co-digestion mixtures. *X_F_X_P_* (*X_F_* − *X_P_*) is the only significant interaction term in the model (*p* < 0.05), although *X_C_X_P_* has a marginal *p*-value of 0.0666. This result shows that the 30-day Y_M_ was more significantly affected by the interaction between FW and PM than those between CM and FW and between CM and PM.

The 30-day SI response surface was also best fitted by a reduced cubic function (Equation (2)):

*Y_S_*_30_ = 1.00*X_C_* + 1.00*X_F_* + 1.00*X_P_* + 0.03*X_C_X_F_* + 0.84*X_C_X_P_* + 0.18*X_F_X_P_* + 1.19*X_C_X_F_*(*X_C_* − *X_F_*) − 0.95*X_F_X_P_*(*X_F_* − *X_P_*) (2)

where *Y_S_*_30_ is the predicted 30-day SI, and *X_C_*, *X_F_*, and *X_P_* are the fractions of CM, FW, and PM in the substrate mixtures (between 0 and 1), respectively. The obtained model showed a good fit to the observed data (*R*^2^ > 0.93, *p* < 0.05), with the coefficient of variation being as low as 3.93% (Table 3). The adequacy-of-precision value was 8.162, which is high enough to ensure the model adequacy (>4) (Table 3). The model was further confirmed for the normal and random distribution of estimation errors. Consequently, the constructed model was reliable for describing the 30-day SI response surface.

**Figure 4:**
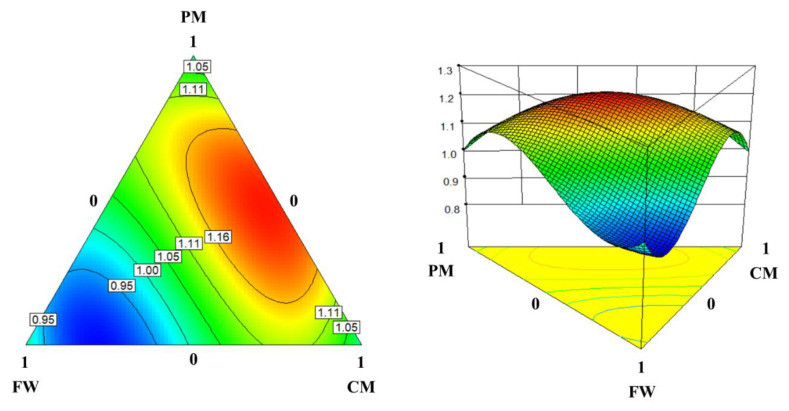
Two- and three-dimensional response surface plots illustrating the effect of the substrate mixing ratio on the 30-day synergy index. Contour colors represent the levels of model response: blue for low and red for high responses.

Aside from the linear effects of the *X_C_*, *X_F_*, and *X_P_*, several interaction terms (i.e., *X_C_X_P_*, *X_C_X_F_* (*X_C_* − *X_F_*), and *X_F_X_P_* (*X_F_* − *X_P_*)) were also significant in the obtained response surface model (*p* < 0.05; Table 4). This result shows that the linear effects of the fractions of CM, FW, and PM in the substrate mixtures and their interactions significantly influence the 30-day SI. Note that the only significant quadratic interaction term, *X_C_X_P_*, had the most significant *p*-value (0.0044) with a high positive coefficient (0.84) (Table 4). This result suggests that mixing CM with PM had a strong synergetic effect on methane production in the co-digestion experiments, as reflected in the 30-day SI response surface plot (Figure 4). The significance of the cubic interactions between CM and FW (i.e., *X_C_X_F_* (*X_C_* − *X_F_*)) and between FW and PM (i.e., *X_F_X_P_* (*X_F_* − *X_P_*)) in the model is also reflected in the response surface, in which the model response changes rapidly (i.e., close contours) along the CM-FW and FW-PM axes. The synergistic effect of the co-digestion is more significant when CM is mixed with PM rather than FW, and the maximum 30-day SI was estimated to be 1.22 for the mixture of 47% CM, 6% FW, and 47% PM. This observation could be explained by the very different characteristics of the substrate wastes (refer to Section 3.1). As seen in Table 1, PM contained relatively high concentrations of free ammonia, lipids, and VFAs, while CM had a high crude fiber content and low VS/TS and soluble COD/total COD ratios. Therefore, co-digesting CM with PM could counteract the undesirable characteristics of each waste, which could lead to digester upset and failure, by blending them into a mixture. The predicted 30-day SI was lower than 1 (i.e., antagonistic effect) under the co-digestion conditions with a higher proportion of FW, and its minimum predicted value was 0.89 at 21% CM: 79% FW. These results agree with the observed data from the BMP experiments, in which only the high-FW co-digestion runs (67% FW; Runs 5, 6, and 11) yielded 30-day SI values lower than 1 (Figure 2B). This result could be attributed to the much more readily biodegradable nature of FW compared to CM and PM (Table 1). It seems that the reduction in methane production from FW had a more significant effect on SI than did the enhanced digestion of CM or PM in high-FW mixtures.

### 3.4. Conclusions and Implications

The overall results show that Y_M_ and SI are affected significantly in different ways by the substrate mixture composition (i.e., the interactions between the substrates). Co-digesting CM with FW and PM proved effective in accelerating the initiation of AD (Figure 2), suggesting that the co-digestion strategy could be applied to promote the start-up of a digester treating CM. No significant enhancement of the ultimate digestibility is anticipated in the co-digestion of high-FW mixtures (30-day SI ≤ 1), whereas the synergistic effect of co-digestion is predicted to be particularly significant in the CM/PM mixtures with little or no FW (30-day SI > 1) (Figure 4). This finding suggests that it is possible to effectively treat the major livestock manures in Korea, namely CM and PM, which are unfavorable for mono-digestion, together through co-digestion.

Important to note is that CM, FW, and PM could be co-digested without a substantial loss of their methane potential (i.e., little antagonistic effect) regardless of the substrate mixing ratio, with the 30-day SI ranging from 0.89 to 1.22. This raises the possibility of treating CM using the spare capacity of existing AD plants handling FW and/or PM. This approach can provide flexibility in CM management while minimizing additional facilities and resources needed. The 30-day SI response surface model predicts an antagonistic effect (30-day SI < 1) only for the co-digestion conditions where the FW fraction is higher than approximately 50% (Figure 4). Therefore, keeping the FW fraction in the substrate mixture less than half of the substrate mixture is advantageous to avoid the possible antagonistic effect, although marginal, and to optimize the benefit from the synergistic effect of co-digestion. The overall results suggest that anaerobic co-digestion with FW and PM can be a viable CM management option in Korea, although further research on the effect of variations in the volume and characteristics of the wastes on the process stability and resilience in continuous mode is needed for practical implementation.

## Figures and Tables

**Table 1:** Physiochemical characteristics of the substrates used in this study.

Parameters	Cattle Manure	Food Waste	Pig Manure
Total solids (g/kg)	310.0 (5.3) ^a^	126.4 (4.5)	70.6 (3.8)
Volatile solids (g/kg)	235.3 (5.8)	120.6 (3.7)	56.5 (1.9)
Volatile-to-total solids ratio (%)	75.9	95.4	80.0
Total suspended solids (g/kg)	280.0 (26.5)	79.5 (2.3)	58.5 (3.5)
Volatile suspended solids (g/kg)	230.0 (31.2)	78.2 (2.0)	46.8 (3.0)
Total COD (g/kg) ^b^	290.8 (25.5)	159.0 (13.6)	134.5 (1.9)
Soluble COD (g/kg)	22.5 (2.2)	52.3 (1.3)	39.4 (0.1)
Soluble-to-total COD ratio (%)	7.7	32.9	29.3
Carbohydrate (%, volatile solids basis)	49.8 (8.1)	59.3 (4.3)	28.3 (4.7)
Protein (%, volatile solids basis)	30.0 (2.4)	20.3 (1.8)	28.3 (6.6)
Lipid (%, volatile solids basis)	0.9 (0.2)	24.2 (1.1)	21.7 (2.1)
Crude fiber (%, volatile solids basis)	32.8 (2.6)	14.7 (2.0)	17.6 (1.2)
Total volatile fatty acids (g COD/kg)	1.5 (0.1)	4.0 (0.6)	22.3 (0.6)
Total nitrogen (g/kg)	9.1 (0.1)	4.4 (0.3)	6.1 (0.3)
Total Kjeldahl nitrogen (g/kg)	12.7 (0.9)	4.3 (0.3)	7.1 (0.6)
Free ammonia nitrogen (g/kg)	1.5 (0.0)	0.4 (0.0)	4.5 (0.1)
Total phosphate (g/kg)	1.0 (0.0)	1.1 (0.4)	1.8 (0.1)
Alkalinity (as g CaCO_3_/kg)	32.7 (0.8)	15.4 (3.4)	13.8 (0.1)
pH	8.0 (0.0)	4.5 (0.0)	7.6 (0.0)
C (%, volatile solids basis)	38.8 (1.1)	53.6 (0.5)	46.4 (0.5)
H (%, volatile solids basis)	4.9 (0.0)	7.9 (0.1)	5.9 (0.1)
O (%, volatile solids basis)	29.3 (0.1)	32.9 (0.2)	34.5 (1.0)
N (%, volatile solids basis)	1.6 (0.0)	3.0 (0.4)	2.1 (0.4)
S (%, volatile solids basis)	<0.01	<0.01	<0.01
C/N ratio	24.3	17.9	22.1

^a^ Standard deviations are given in parentheses; ^b^ COD, chemical oxygen demand.

**Table 2:** Composition and characteristics of substrate mixtures.

Run	Mixing Ratio (Total Solids Basis)			C/N Ratio ^a^	Volatile-to-Total Solids Ratio (%) ^a^	Soluble-to-Total COD Ratio (%) ^a,b^
	Cattle Manure	Food Waste	Pig Manure			
1	1.00	0.00	0.00	24.3	75.9	7.7
2	0.00	1.00	0.00	17.9	95.4	32.9
3	0.00	0.00	1.00	22.1	80.0	29.3
4	0.67	0.33	0.00	20.8	79.2	13.1
5	0.33	0.67	0.00	19.0	84.7	20.9
6	0.00	0.67	0.33	18.8	92.0	31.8
7	0.00	0.33	0.67	20.1	87.3	30.6
8	0.67	0.00	0.33	23.4	76.3	11.8
9	0.33	0.00	0.67	22.7	77.2	18.1
10	0.67	0.17	0.17	21.9	77.8	12.5
11	0.17	0.67	0.17	18.9	87.4	25.5
12	0.17	0.17	0.67	21.2	80.9	23.5
13	0.33	0.33	0.33	20.4	81.3	19.5

^a^ Calculated based on the characteristics and mixing ratios of the substrates; ^b^ COD, chemical oxygen demand.

**Table 3:** Statistical significance of the response surface models.

Response	Standard Deviation	Coefficient of Variation (%)	*R* ^2^	*p*-Value	Adequacy of Precision
**Methane Yield**	21.11	6.15	0.9789	<0.0001	27.844
**Synergy Index**	0.042	3.93	0.9307	0.012	8.162

**Table 4:** Statistical significance of the model coefficients.

Methane Yield Model					Synergy Index Model				
Terms ^a^	Coefficient	Standard Error	*F*-Value	*p*-Value	Terms	Coefficient	Standard Error	*F*-Value	*p*-Value
**Linear Mixture**			157.00	<0.0001	Linear mixture			12.96	0.0105
***X_C_X_P_***	185.16	85.32	4.71	0.0666	*X_C_X_F_*	0.03	0.17	0.04	0.8547
***X_F_X_P_***	96.62	85.00	1.29	0.2931	*X_C_X_P_*	0.84	0.17	24.17	0.0044
***X_F_X_P_*** **(*X_F_* − *X_P_*)**	−460.79	177.34	6.75	0.0355	*X_F_X_P_*	0.18	0.17	1.10	0.3433
					*X_C_X_F_*(*X_C_* − *X_F_*)	1.19	0.35	11.25	0.0202
					*X_F_X_P_*(*X_F_* − *X_P_*)	−0.95	0.35	7.15	0.0442

^a^*X_C_*, *X_F_*, and *X_P_* are the fractions of CM, FW, and PM in the substrate mixtures (between 0 and 1), respectively. Refer to Equations (1) and (2).
